# Supplementary material for: Hybridizing anomalous Nernst effect in artificially tilted multilayer based on magnetic topological material
Source: Nat Commun. 2024 Nov 14;15:9643. doi: 10.1038/s41467-024-53723-2 (PMC11564512; doi:10.1038/s41467-024-53723-2)
Supplement: Supplementary file 1 — Supplementary Information [file 41467_2024_53723_MOESM1_ESM.pdf]

Supplementary Information for "Hybridizing anomalous Nernst effect in artificially tilted multilayer based on magnetic topological material"

Takamasa Hirai[1]*, Fuyuki Ando[1], Hossein Sepehri-Amin[1], and Ken-ichi Uchida[1,2]*

[1]National Institute for Materials Science, Tsukuba 305-0047, Japan.
[2]Department of Advanced Materials Science, Graduate School of Frontier Sciences, The University of Tokyo, Kashiwa 277-8561, Japan.
*e-mail: HIRAI.Takamasa@nims.go.jp; UCHIDA.Kenichi@nims.go.jp

Table of contents

Supplementary Note 1 | Calculation of transport properties in artificially tilted multilayers.

Supplementary Note 2 | Anisotropy of electric and thermoelectric transport properties in sintered $Bi_{0.2}Sb_{1.8}Te_3$ and Bi_2Te_3.

Supplementary Table 1 | Transport properties of Co_2MnGa, $Bi_{0.2}Sb_{1.8}Te_3$, and Bi_2Te_3 slabs.

Supplementary Fig. 1 | Structural and chemical characterization in Co_2MnGa-based multilayers.

Supplementary Fig. 2 | Measurements of the Nernst effect.

Supplementary Fig. 3 | Simulation of transverse thermopower and electrical/thermal conductivity for Co_2MnGa/Bi_2Te_3 ATML.

Supplementary Fig. 4 | Transverse thermoelectric conversion in Co_2MnGa/Bi_2Te_3 ATML at zero magnetic field.

Supplementary Fig. 5 | Contribution of transverse magneto-thermoelectric effects in Co_2MnGa/Bi_2Te_3 ATML in cross-section configuration.

Supplementary Fig. 6 | Contribution of transverse magneto-thermoelectric effects in Co_2MnGa/Bi_2Te_3 ATML in top-side configuration.

Supplementary Fig. 7 | Simulation of dimensionless figure of merit for transverse thermoelectric generation with hybridized ODSE and ANE in ATML.

Supplementary Fig. 8 | Dependence of electrical and thermoelectric transport properties of sintered $Bi_{0.2}Sb_{1.8}Te_3$ and Bi_2Te_3 on pressing direction.

Supplementary References

Supplementary Note 1 | Calculation of transport properties in artificially tilted multilayers.

Referring to ref. 1 and 2 in Supplementary Information, the thermoelectric, electrical, thermal transport parameters of magnetic/thermoelectric multilayers in the direction parallel ($S_{||}$, $\sigma_{||}$, and $\kappa_{||}$) and perpendicular ($S_{\perp}$, $\sigma_{\perp}$, and $\kappa_{\perp}$) to the stacking plane are formulated using the following Supplementary Equations (1)-(3) on the assumption that the interfacial electrical and thermal resistances at boundaries between magnetic and thermoelectric layers are negligibly small:

$$S_{||} = \frac{R\sigma_{\mathrm{M}}S_{\mathrm{S,M}} + (1-R)\sigma_{\mathrm{TE}}S_{\mathrm{S,TE}}}{R\sigma_{\mathrm{M}} + (1-R)\sigma_{\mathrm{TE}}}, \quad S_{\perp} = \frac{R\kappa_{\mathrm{TE}}S_{\mathrm{S,M}} + (1-R)\kappa_{\mathrm{M}}S_{\mathrm{S,TE}}}{R\kappa_{\mathrm{TE}} + (1-R)\kappa_{\mathrm{M}}} \tag{1}$$

$$\sigma_{||} = R\sigma_{\mathrm{M}} + (1-R)\sigma_{\mathrm{TE}}, \quad \sigma_{\perp} = \frac{\sigma_{\mathrm{M}}\sigma_{\mathrm{TE}}}{(1-R)\sigma_{\mathrm{M}} + R\sigma_{\mathrm{TE}}} \tag{2}$$

$$\kappa_{||} = R\kappa_{\mathrm{M}} + (1-R)\kappa_{\mathrm{TE}}, \quad \kappa_{\perp} = \frac{\kappa_{\mathrm{M}}\kappa_{\mathrm{TE}}}{(1-R)\kappa_{\mathrm{M}} + R\kappa_{\mathrm{TE}}} \tag{3}$$

Here, $S_{\mathrm{S,M}}$ ($S_{\mathrm{S,TE}}$) is the Seebeck coefficient, σ_{M} (σ_{TE}) the electrical conductivity, and κ_{M} (κ_{TE}) the thermal conductivity of the magnetic (thermoelectric) component and R [$= t_{\mathrm{M}}/(t_{\mathrm{M}}+t_{\mathrm{TE}})$] is the thickness ratio with t_{M} (t_{TE}) being the thickness of the magnetic (thermoelectric) component. When the magnetic/thermoelectric stacks are rotated with the tilt angle θ to form artificially tilted multilayers (ATMLs), the transverse thermopower due to the off-diagonal Seebeck effect (ODSE), S_{OD}, and the electrical and thermal conductivities orthogonal to each other (σ_{xx} and κ_{yy}) are respectively expressed as

$$S_{\mathrm{OD}} = \left(S_{\perp} - S_{||}\right)\sin\theta\cos\theta \tag{4}$$

$$\sigma_{xx} = \frac{\sigma_{||}\sigma_{\perp}}{\sigma_{||}\sin^2\theta + \sigma_{\perp}\cos^2\theta} \tag{5}$$

$$\kappa_{yy} = \kappa_{||}\sin^2\theta + \kappa_{\perp}\cos^2\theta \tag{6}$$

With these parameters, the dimensionless figure of merit for ODSE $z_{\mathrm{OD}}T$ with T being the absolute temperature is defined as

$$z_{\mathrm{OD}}T = \frac{S_{\mathrm{OD}}^2\sigma_{xx}}{\kappa_{yy}}T \tag{7}$$

Supplementary Note 2 | Anisotropy of electric and thermoelectric transport properties in sintered $Bi_{0.2}Sb_{1.8}Te_3$ and Bi_2Te_3.

From the sintered cylindrical Bi_2Te_3 and $Bi_{0.2}Sb_{1.8}Te_3$ ingots with a diameter of 20 mm and a height of 15 mm, two rectangular slabs, named "Slab 1" and "Slab 2", with a size of ~12 × 3 × 1 mm^3 were cut out using the diamond wire saw, where the longitudinal direction of Slab1 (Slab2) was along the diameter (height) direction of the ingot, i.e., perpendicular (parallel) to the pressing direction during spark plasma sintering (SPS) (Supplementary Fig. 7a). Supplementary Fig. 7b shows the Seebeck coefficient S_{S} and electrical conductivity σ of Slabs 1 and 2 for Bi_2Te_3 and $Bi_{0.2}Sb_{1.8}Te_3$. In both the Bi_2Te_3 and $Bi_{0.2}Sb_{1.8}Te_3$ slabs, there was little difference in S_{S} between Slabs 1 and 2, while the magnitude of σ of Slab 2 was clearly smaller than that of Slab 1 even though our $Bi_{0.2}Sb_{1.8}Te_3$ and Bi_2Te_3 slabs are polycrystalline. The behavior of isotropic S_{S} and anisotropic σ in Bi-Sb-Te and Bi-Te prepared by SPS has been also reported in the previous study; it is not due to their intrinsic band structure but due to the particle shape of the matrix powder[3]. However, the observed anisotropy of σ has little impact on the transport properties of our ATMLs; the value of dimensionless figure of merit for the off-diagonal Seebeck effect calculated with the parameters for Slab 1 was almost the same as that for Slab 2 (Supplementary Fig. 7c,d and Supplementary Note 1 for calculation details).

Supplementary Table 1 | Transport properties of Co_2MnGa, $Bi_{0.2}Sb_{1.8}Te_3$, and Bi_2Te_3 slabs. The value of σ, thermal conductivity κ, thermal diffusivity D, specific heat C, density ρ, S_S, ordinary or anomalous Nernst coefficient S_N at the magnetic field $\mu_0|H|$ = 0 and 0.8 T with μ_0 and H being the vacuum permeability and magnitude of the magnetic field, respectively.

	Co_2MnGa		$Bi_{0.2}Sb_{1.8}Te_3$		Bi_2Te_3	
	$\mu_0\|H\|$ = 0 T	0.8 T	0 T	0.8 T	0 T	0.8 T
σ (×10^5 S/m)	7.99	8.00	1.28	1.28	1.83	1.83
κ (W/mK)	18.7	n/a	1.2	n/a	1.5	n/a
D (×10^{-6} m^2/s)	5.17	n/a	0.86	n/a	1.14	n/a
C (J/gK)	0.44	n/a	0.22	n/a	0.18	n/a
ρ (×10^6 g/m^3)	8.33	n/a	6.37	n/a	7.12	n/a
S_S (μV/K)	−32.1 ± 0.5	−32.0 ± 0.5	170.6 ± 0.5	170.5 ± 0.5	−110.3 ± 0.7	−109.7 ± 0.6
S_N (μV/K)	n/a	6.9 ± 0.2	n/a	−1.7 ± 0.3	n/a	−1.4 ± 0.1

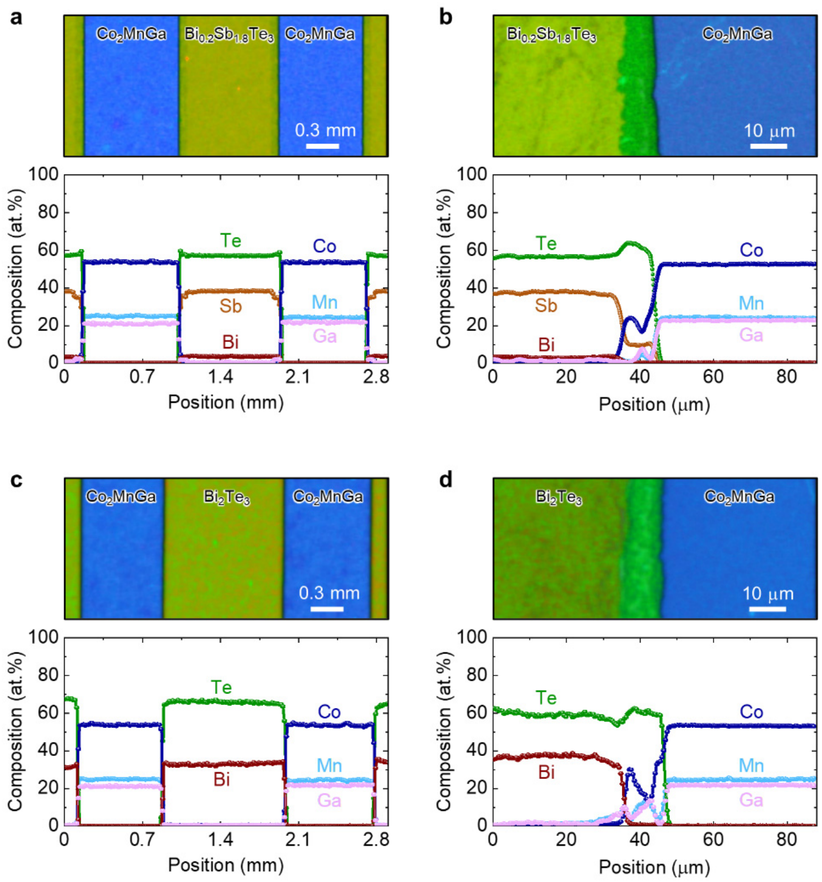

Supplementary Fig. 1 | Structural and chemical characterization in Co_2MnGa-based multilayers. a,b Cross-sectional scanning electron microscopy with energy-dispersive X-ray spectroscopy mapping images for the $Co_2MnGa/Bi_{0.2}Sb_{1.8}Te_3$ multilayer with low (**a**) and high (**b**) magnification. Line profiles of the atomic composition across the stacking direction in the mapping images are also shown. **c,d** Results for the Co_2MnGa/Bi_2Te_3 multilayer.

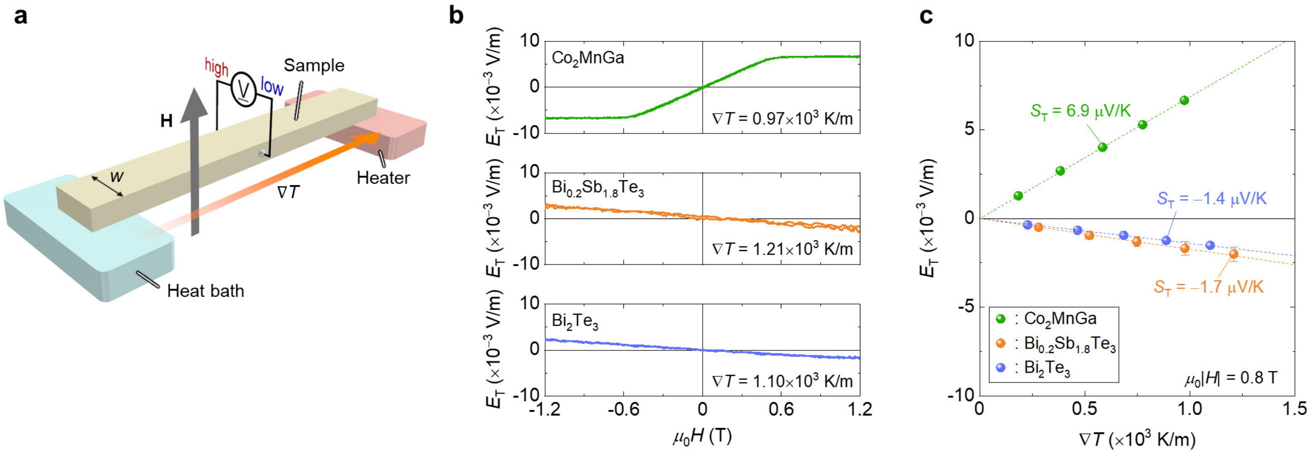

Supplementary Fig. 2 | Measurements of the Nernst effect. a Schematic of the set-up for measuring transverse thermoelectric voltage V_T due to the ordinary or anomalous Nernst effect, where **H**, ∇T, and w denote the magnetic field vector, temperature gradient, and sample width, respectively. **b** H dependence of the transverse electric field E_T (= V_T/w) for the Co_2MnGa, $Bi_{0.2}Sb_{1.8}Te_3$, and Bi_2Te_3 slabs at $\nabla T = 0.97 \times 10^3$, 1.21×10^3, and 1.10×10^3 K/m, respectively. The value of ∇T was measured by the infrared camera (see Methods). **c** ∇T dependence of E_T for the Co_2MnGa, $Bi_{0.2}Sb_{1.8}Te_3$, and Bi_2Te_3 slabs at $\mu_0|H| = 0.8$ T. Dotted lines show the results of linear fitting to determine S_N.

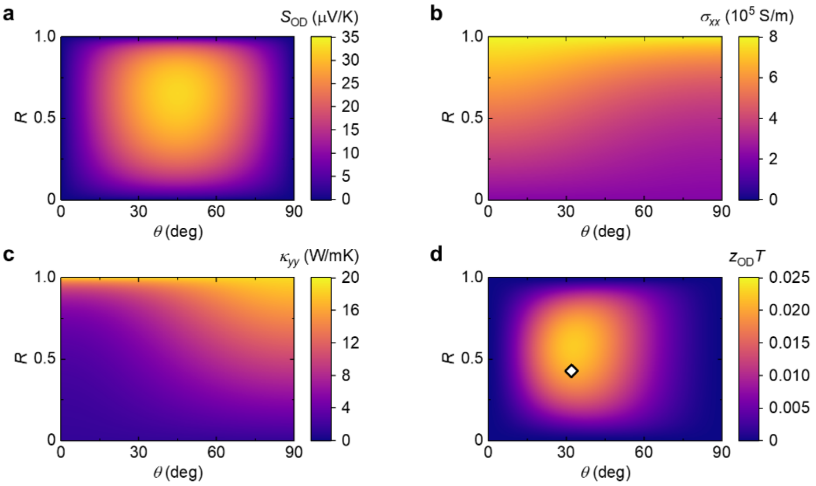

Supplementary Fig. 3 | Simulation of transverse thermopower and electrical/thermal conductivity for Co_2MnGa/Bi_2Te_3 ATML. a-d Contour maps depicting S_{OD} (**a**), σ_{xx} (**b**), κ_{yy} (**c**), and $z_{OD}T$ at zero magnetic field and $T = 300$ K (**d**) as functions of θ and R. The open diamond symbol in (**d**) represents the θ (= 32° ± 1°) and R (= 0.43) values.

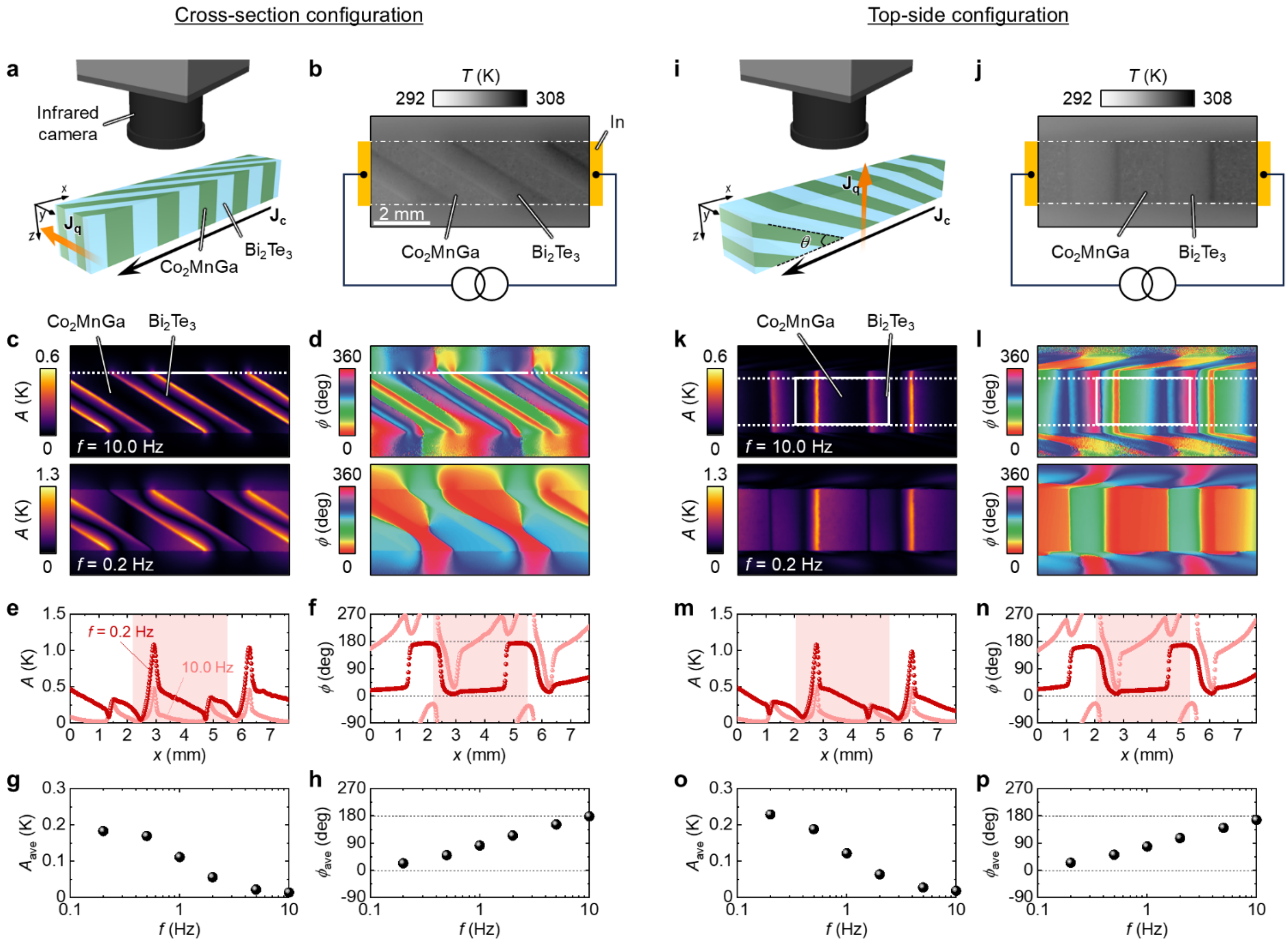

Supplementary Fig. 4 | Transverse thermoelectric conversion in Co_2MnGa/Bi_2Te_3 ATML at zero magnetic field. a Schematic of the sample structure in the cross-section configuration. **b** Steady-state temperature image during the LIT measurement in the cross-section configuration. **c,d** Lock-in amplitude A (**c**) and phase ϕ (**d**) images at the lock-in frequency f = 10.0 and 0.2 Hz. **e,f** x-directional A (**e**) and ϕ (**f**) profiles along the white dotted lines in the top panels of **c** and **d**, respectively. **g,h** f dependence of the averaged lock-in amplitude A_{ave} (**g**) and phase ϕ_{ave} (**h**) values over one Co_2MnGa/Bi_2Te_3 unit. **i-p** Results for the top-side configuration. The A_{ave} and ϕ_{ave} values in **g** and **h** (**o** and **p**) were estimated by averaging A and ϕ signals in the areas defined by the white rectangles in **c** and **d** (**k** and **l**), corresponding to the blue shaded area in **e** and **f** (**m** and **n**), respectively. In all the LIT measurements, a square-wave-modulated charge current with an amplitude of 1 A and zero offset was applied.

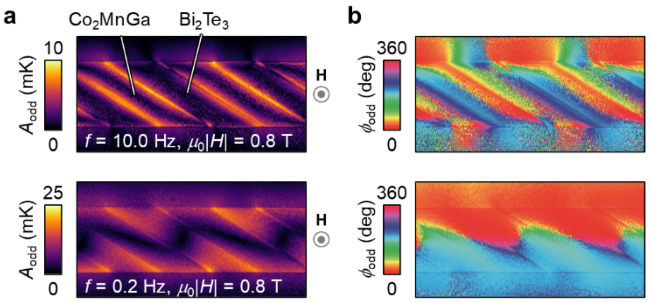

Supplementary Fig. 5 | Contribution of transverse magneto-thermoelectric effects in Co_2MnGa/Bi_2Te_3 ATML in cross-section configuration. a,b H-odd-dependent component of the lock-in amplitude A_{odd} (**a**) and phase ϕ_{odd} (**b**) images at $\mu_0|H|$ = 0.8 T and f = 10.0 and 0.2 Hz in Co_2MnGa/Bi_2Te_3 ATML. Here, the magnetic field **H** with the magnitude of H was applied along the vertical direction (z axis in Supplementary Fig. 4a) of the sample and μ_0 is the vacuum permeability.

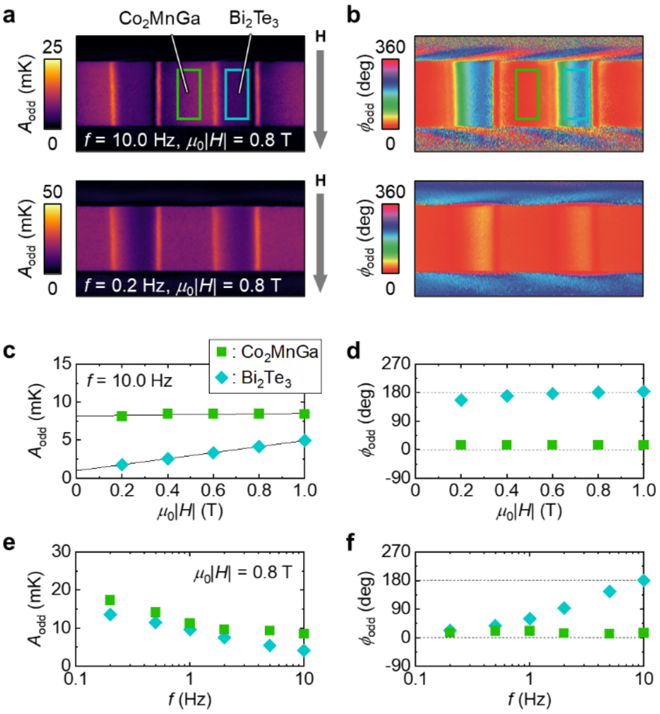

Supplementary Fig. 6 | Contribution of transverse magneto-thermoelectric effects in Co_2MnGa/Bi_2Te_3 ATML in top-side configuration. a,b A_{odd} (**a**) and ϕ_{odd} (**b**) images at $\mu_0|H|$ = 0.8 T and f = 10.0 and 0.2 Hz in Co_2MnGa/Bi_2Te_3 ATML. Here, **H** was applied along the horizontal short direction of the sample (y axis in Supplementary Fig. 4i). **c,d** $|H|$ dependence of A_{odd} (**c**) and ϕ_{odd} (**d**) at f = 10.0 Hz. Solid lines represent the results of linear fitting. **e,f** f dependence of A_{odd} (**e**) and ϕ_{odd} (**f**) at $\mu_0|H|$ = 0.8 T. The data points in **c-f** were obtained by averaging the temperature modulation signal in the area defined by the green and blue rectangles in **a** and **b** for the Co_2MnGa and Bi_2Te_3 areas, respectively.

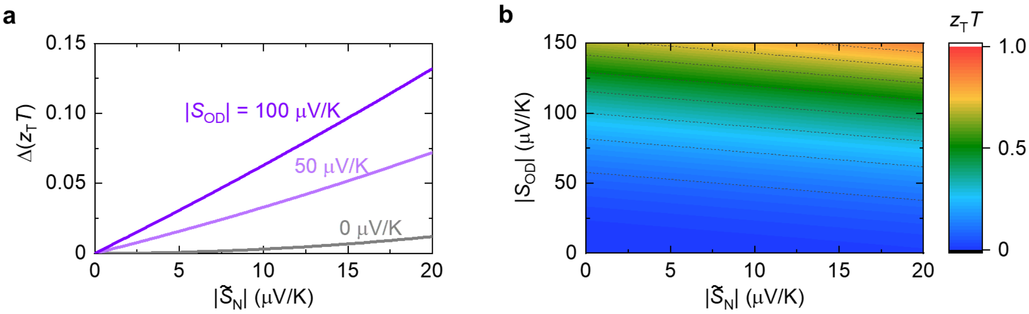

Supplementary Fig. 7 | Simulation of dimensionless figure of merit for transverse thermoelectric generation with hybridized ODSE and ANE in ATML. a Dependence of the absolute value of $\tilde{S}_N$ on the magneto-thermoelectric modulation of the dimensionless figure of merit for hybrid transverse magneto-thermoelectric conversion $z_T T$, i.e., $\Delta(z_T T) = \tilde{S}_N^2 + 2|S_{OD}\tilde{S}_N|$, at various $|S_{OD}|$ values, where $\tilde{S}_N$ is the effective anomalous Nernst coefficient in ATML [see Equation (4) in the main text]. **b** Contour maps of $z_T T$ as functions of $|\tilde{S}_N|$ and $|S_{OD}|$. Here, σ_{xx} = 3.3×10^5 S/m and κ_{yy} = 3.7 W/mK obtained for $Co_2MnGa/Bi_{0.2}Sb_{1.8}Te_3$ ATML were used for estimating $\Delta(z_T T)$ and $z_T T$.

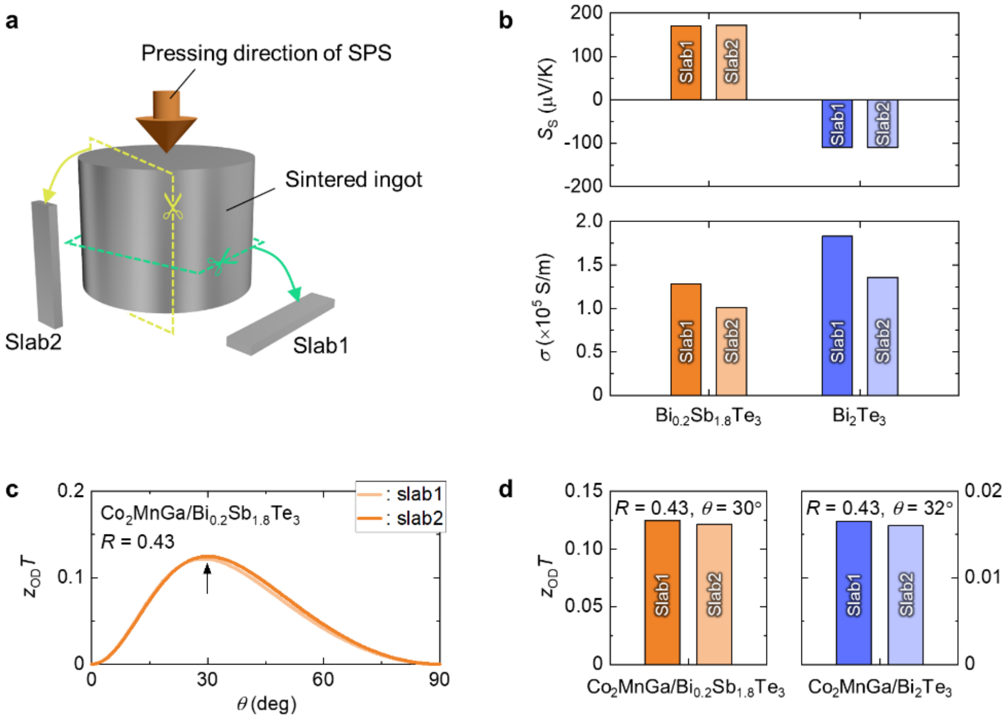

Supplementary Fig. 8 | Dependence of electrical and thermoelectric transport properties of sintered $Bi_{0.2}Sb_{1.8}Te_3$ and Bi_2Te_3 on pressing direction. a Schematic of the preparation of $Bi_{0.2}Sb_{1.8}Te_3$ and Bi_2Te_3 slabs. **b** S_S and σ of Slabs 1 and 2 for $Bi_{0.2}Sb_{1.8}Te_3$ and Bi_2Te_3. **c** θ dependence of simulated $z_{OD}T$ for $Co_2MnGa/Bi_{0.2}Sb_{1.8}Te_3$ ATML at R = 0.43. The black arrow shows $z_{OD}T$ at θ of our $Co_2MnGa/Bi_{0.2}Sb_{1.8}Te_3$ ATML (= 30°). The calculated value of $z_{OD}T$ is not changed by the difference in transport properties between Slabs 1 and 2. **d** $z_{OD}T$ at R = 0.43 and θ = 30° (32°) for $Co_2MnGa/Bi_{0.2}Sb_{1.8}Te_3$ (Co_2MnGa/Bi_2Te_3) ATML.

Supplementary References

1. Goldsmid, H. J. Application of the transverse thermoelectric effects. *J. Electron. Mater.* **40**, 1254-1259 (2011).
2. Ando, F. et al. Multifunctional composite magnet for practical transverse thermoelectrics. Preprint at https://arxiv.org/abs/2402.18019 (2024).
3. Kim, D. H. et al. Influence of powder morphology on thermoelectric anisotropy of spark-plasma-sintered Bi-Te-based thermoelectric materials. *Acta Mater.***59**, 405-411 (2011).
